# A remarkable short-snouted horned dinosaur from the Late Cretaceous (late Campanian) of southern Laramidia

**DOI:** 10.1098/rspb.2013.1186

**Published:** 2013-09-07

**Authors:** Scott D. Sampson, Eric K. Lund, Mark A. Loewen, Andrew A. Farke, Katherine E. Clayton

**Affiliations:** 1 Denver Museum of Nature and Science, 2001 Colorado Boulevard, Denver, CO 80205, USA; 2 Department of Geology and Geophysics, University of Utah, 115 S 1460 East, Salt Lake City, UT 84112, USA; 3 Natural History Museum of Utah, University of Utah, 301 Wakara Way, Salt Lake City, UT 84108, USA; 4 Department of Biomedical Sciences, Ohio University, Heritage College of Osteopathic Medicine, Athens, OH 45701, USA; 5 Raymond M. Alf Museum of Paleontology, 1175 West Baseline Road, Claremont, CA 91711, USA

**Keywords:** Laramidia, Dinosauria, Ceratopsidae, Centrosaurinae, Kaiparowits Formation, *Nasutoceratops titusi*

## Abstract

The fossil record of centrosaurine ceratopsids is largely restricted to the northern region of western North America (Alberta, Montana and Alaska). Exceptions consist of single taxa from Utah (*Diabloceratops*) and China (*Sinoceratops*), plus otherwise fragmentary remains from the southern Western Interior of North America. Here, we describe a remarkable new taxon, *Nasutoceratops titusi* n. gen. et sp., from the late Campanian Kaiparowits Formation of Utah, represented by multiple specimens, including a nearly complete skull and partial postcranial skeleton. Autapomorphies include an enlarged narial region, pneumatic nasal ornamentation, abbreviated snout and elongate, rostrolaterally directed supraorbital horncores. The subrectangular parietosquamosal frill is relatively unadorned and broadest in the mid-region. A phylogenetic analysis indicates that *Nasutoceratops* is the sister taxon to *Avaceratops*, and that a previously unknown subclade of centrosaurines branched off early in the group's history and persisted for several million years during the late Campanian. As the first well-represented southern centrosaurine comparable in age to the bulk of northern forms, *Nasutoceratops* provides strong support for the provincialism hypothesis, which posits that Laramidia—the western landmass formed by inundation of the central region of North America by the Western Interior Seaway—hosted at least two coeval dinosaur communities for over a million years of late Campanian time.

## 1. Introduction

During the Late Cretaceous, elevated global sea levels subdivided North America into eastern and western landmasses—Appalachia and Laramidia, respectively—for about 27 million years (approx. 95–68 Myr ago). Laramidia witnessed the greatest radiation of Mesozoic dinosaurs documented to date [1], with Ceratopsidae—an assemblage of large-bodied ornithischian herbivores bearing signature skull ornamentations—being the most speciose clade. Within Ceratopsidae, centrosaurines are known overwhelmingly from the northern region of Laramidia, with 15 of 17 named species recovered from Alberta, Montana and Alaska. The two exceptions are *Sinoceratops* from the Campano-Maastrichtian of China [2] and *Diabloceratops* from the early Campanian of Utah [3]. Additional, non-diagnostic centrosaurine material is known from the Fort Crittendon Formation of New Mexico [4], the Menefee Formation of New Mexico [5] and the Cerro del Pueblo Formation of Mexico [6].

Since 2000, a collaborative, multi-institutional team working in southern Utah's Grand Staircase-Escalante National Monument has unearthed a previously unknown dinosaur assemblage from the late Campanian Kaiparowits Formation [1]. A total of 16 taxa have been identified from this unit, including the hadrosaurine *Gryposaurus monumentensis* [7], the oviraptorosaurid *Hagryphus giganteus* [8], the tyrannosaurid *Teratophoneus curriei* [9], the troodontid *Talos sampsoni* [10] and a pair of chasmosaurine ceratopsids, *Utahceratops gettyi* and *Kosmoceratops richardsoni* [11,12]. Here, we report the discovery of a remarkable new long-horned centrosaurine from the Kaiparowits Formation, to our knowledge the first late Campanian member of this clade described from southern Laramidia. This taxon sheds light on the evolution of Centrosaurinae and offers key insights into Laramidian dinosaur provincialism.

## 2. Systematic palaeontology

Dinosauria Owen, 1842 *sensu* Padian and May 1993

 Ornithischia Seeley, 1887 *sensu* Sereno 1998

 Ceratopsia Marsh, 1890 *sensu* Dodson, 1997

 Ceratopsidae Marsh, 1888 *sensu* Sereno 1998

 Centrosaurinae Lambe, 1915 *sensu* [13]

 *Nasutoceratops titusi* n. gen. et sp.

 urn:lsid:zoobank.org:act:F9997290-2618-4C95-9D46-7EED00C99916

### (a) Etymology

From the Latin *nasutus*, meaning ‘large-nosed’ and the Latinized Greek *ceratops*, meaning ‘horned-face’; *titusi*, honouring Alan Titus, palaeontologist at Grand Staircase-Escalante National Monument, for his exemplary efforts assisting palaeontological fieldwork in the Monument.

### (b) Material

Natural History Museum of Utah (UMNH) VP 16800, holotype consisting of a mostly complete, articulated 1.8 m long skull plus postcranial elements: syncervical, three fragmentary dorsal vertebrae, associated left forelimb and fragmentary right forelimb. Referred materials consist of UMNH VP 19466—a disarticulated adult skull including a partial premaxilla, maxilla and nasal—and UMNH VP 19469, an isolated squamosal.

### (c) Locality and horizon

**Figure 1.**
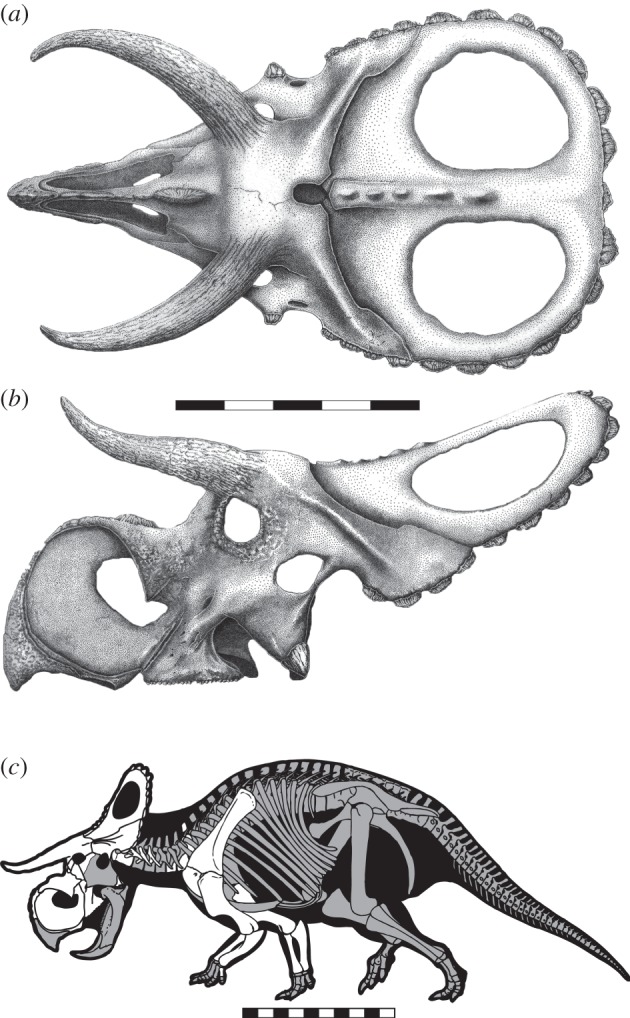
*Nasutoceratops titusi*, n. gen et. sp., skull reconstruction in (*a*) dorsal and (*b*) lateral views. (*c*) Skeletal reconstruction with elements presently known in white. (*a*,*b*) Scale bars, 50 cm and (*c*) 1 m.

UMNH VP 16800 was collected in 2006 from UMNH VP Locality 940 [14] within Grand Staircase-Escalante National Monument, southern Utah, USA. Stratigraphically, *Nasutoceratops* occurs within the middle unit (approx. 250–320 m) of the Upper Campanian Kaiparowits Formation, dated to the late Campanian, approximately between 75.51 and 75.97 Ma (figure 1; [15,16]; recalibrated in Roberts *et al*. [17]). Detailed locality information for *Nasutoceratops* on file at the Natural History Museum of Utah, Salt Lake City, UT.

### (d) Diagnosis

Centrosaurine ceratopsid bearing the following autapomorphies: hypertrophied narial region (approx. 75% preorbital skull length); caudal portion of fused nasals occupied by internal pneumatic cavity; uniquely enlarged premaxillary contact of maxilla; double faceted, medially directed flange on maxilla and rostrolaterally directed, rostrally curved and apically twisted supraorbital horncores; *Nasutoceratops* can also be distinguished by a unique suite of synapomorphies, including: low, transversely narrow, rostrocadually elongate nasal horncore; pronounced dorsolateral ridge on squamosal; subcircular parietosquamosal frill widest near mid-region; simple, crescentic episquamosals and epiparietals; and the presence of a midline epiparietal.

## 3. Description

The lower level taxonomy of ceratopsid dinosaurs has been based almost exclusively on craniofacial characters. Thus, the abbreviated description of *Nasutoceratops* below is limited to diagnostic aspects of skull anatomy.

### (a) Narial region

The bony anatomy of the narial region of centrosaurines, although derived relative to other dinosaurs, tends to be conservative within the clade. Yet, this region is unique in several respects in *Nasutoceratops*. Overall, the preorbital facial skeleton is relatively short rostrocaudally, comprising less of the total skull length than in any other ceratopsid (see the electronic supplementary material). Yet, the narial region, dominated by the premaxilla and ectonaris, is hypertrophied, comprising approximately 75% of preorbital skull length. The front of the snout is also expanded dorsally, resulting in an ‘inflated’ appearance compared with other centrosaurine taxa. Associated with expansion of the narial region is an enlarged premaxillary septum, also more extensive than in other centrosaurines.

The ascending ramus of the maxilla is rostrocaudally abbreviated and the maxillary body is dorsoventrally expanded, both features presumably related to the premaxillary expansion. The conformation of this morphology is reflected in the steeply inclined ascending ramus; centrosaurines typically possess a more caudally projected, rostrocaudally thickened ascending ramus. The holotype maxilla preserves 22 maxillary tooth positions, and the referred *Nasutoceratops* specimen UMNH VP 19466 includes a complete maxilla bearing 29 alveoli. The teeth are typical of centrosaurines. The maxillary tooth row of *Nasutoceratops* is displaced ventrally, much as in *Albertaceratops*, *Avaceratops*, *Diabloceratops* and many non-ceratopsid neoceratopsians. The premaxillary contact surface on the maxilla is exceptionally broad transversely, forming a deep concavity ventrally and becoming shallower dorsally. A double faceted, medially directed flange of the maxilla slots into premaxilla, forming a greater contribution to the hard palate than in related forms.

Like the maxilla, the nasal of *Nasutoceratops* is somewhat foreshortened relative to the condition in more derived centrosaurines. The nasal horncore is relatively low, rostrocaudally elongate and bladelike (transversely narrow), situated dorsal to the endonaris. The bulk of derived centrosaurines exhibit more elaborate forms of nasal ornamentation, including large horncores of varying orientation (e.g. *Centrosaurus*, *Einiosaurus* and *Styracosaurus*) and pachyostotic bosses (*Achelousaurus* and *Pachyrhinosaurus*). The relatively diminutive horncore of *Nasutoceratops*, by contrast, more closely resembles that of the basal centrosaurine *Albertaceratops* [18]. Both of the known nasal specimens of *Nasutoceratops* exhibit well-developed, caudally positioned internal cavities within the body of the nasal caudal to the horncore. These cavities are here interpreted to represent pneumatic excavations from the paranasal region. Pneumatic nasals are unknown in any other ceratopsid, and this feature is here regarded to be autapomorphic for the new Utah taxon.

### (b) Circumorbital region

The supraorbital horncores of *Nasutoceratops* are highly distinctive, being exceptionally elongate, rostrodorsally oriented and twisted distally. Within Centrosaurinae, supraorbital horncores tend to be relatively short, the only exceptions being the basal taxa *Diabloceratops*, *Avaceratops* and *Albertaceratops*. Given that the sister taxon to Ceratopsidae, *Zuniceratops*, also exhibits elongate supraorbital horncores [19], this feature is probably symplesiomorphic for centrosaurines. The horncores of *Nasutoceratops* are curved throughout their lengths, transitioning in orientation from rostrolateral to rostromedial at the tips, with the distal one-third exhibiting pronounced lateral torsion. This torsion combines dorsal curvature and twisting of the distal horncore, marked by longitudinal grooves along the external surface. In the holotype, UMNH VP 16800, the supraorbital horncores are relatively and absolutely the longest of any centrosaurine, spanning approximately 40% of total skull length and extending rostrally almost to the tip of the snout.

Caudal to the nasal horncore and immediately rostral to the orbits, the dorsum of the skull inclines dorsally to form a pronounced ‘forehead’ and vaulted skull roof composed largely of nasals, prefrontals, palpebrals and frontals. Whereas the majority of centrosaurines, and ceratopsids generally, possess a relatively flat skull roof between the facial skeleton and the cranium, the steeply inclined preorbital region of *Nasutoceratops* closely resembles that of the centrosaurines *Albertaceratops* and *Diabloceratops* [3,18], as well as the chasmosaurines *Kosmoceratops*, *Utahceratops* and *Pentaceratops* [11,12].

Only a small dorsal portion of the jugal is preserved on UMNH VP 16800. However, the *Nasutoceratops* holotype does include a well-preserved epijugal ossification. Whereas large epijugals are typical of chasmosaurine ceratopsids, centrosaurines tend to possess relatively small accessory ossifications on the distal jugal. By contrast, the epijugal of *Nasutoceratops* more closely resembles that of the basal centrosaurine *Diabloceratops*, in which this element is also strongly developed. The UMNH VP 16800 epijugal—relatively and absolutely the largest example known among centrosaurines—is approximately trihedral, with a flattened rostral surface. Given the distribution of large epijugals among chasmosaurines, some non-ceratopsid neoceratopsians (e.g. *Protoceratops*), and basal centrosaurines, this feature is probably symplesiomorphic for Ceratopsidae.

### (c) Parietosquamosal frill

In overall conformation, the parietosquamosal frill of *Nasutoceratops* resembles that of most centrosaurines (e.g. *Centrosaurus*, *Einiosaurus* and *Achelousaurus*), bearing a transversely convex and rostrocaudally concave dorsal surface. A large, oval parietal fenestra is present on either side, with the long axis oriented rostrocaudally. The *Nasutoceratops* frill is subrectangular as viewed dorsally, with the broadest point occurring in the mid-region. Total frill length, estimated at 610 mm in UMNH VP 16800, is approximately equal to basal skull length.

The squamosal is poorly preserved in UMNH VP 16800, but a referred, mostly complete specimen, UMNH VP 19469, has been recovered from the same stratigraphic interval of the Kaiparowits Formation. The latter specimen demonstrates that the squamosal of *Nasutoceratops* closely resembles that of other centrosaurines in being relatively short rostrocaudally with a stepped caudomedial margin [13]. However, *Nasutoceratops* differs in possessing a pronounced ridge on the dorsolateral surface, which, in UMNH VP 19469, can be seen to extend most of the element's length. Other centrosaurines (e.g. *Avaceratops* and *Albertaceratops*) possess raised bumps in this area, but a fully formed, elongate ridge in *Nasutoceratops* extends approximately three times the length of the laterotemporal fenestra. This distinctive feature is otherwise known only on a centrosaurine squamosal (NMMNH P34906) from the Fort Crittenden Formation of Arizona [4], and another example (NMMNH P25052) from the Menefee Formation of New Mexico [5].

Among centrosaurines, the parietal of *Nasutoceratops* and *Avaceratops* is distinctive in lacking both a median embayment along the caudal margin and well-developed marginal ornamentations. In most centrosaurines, the parietal margin exhibits a unique suite of ‘spikes’ (e.g. *Styracosaurus* and *Einiosaurus*), ‘hooks’ (*Centrosaurus* and *Coronosaurus*) and/or ‘horns’ (e.g. *Pachyrhinosaurus*). These structures, formed largely by accessory ossifications (epiparietals), occur even in basal members of the clade (e.g. *Diabloceratops* [3], *Xenoceratops* [20] and *Albertaceratops* [18]). Although *Nasutoceratops* possesses marginal undulations topped with epiparietals, the latter are relatively small and uniformly crescentic, lacking any prominent spikes or hooks. Parietal conformation is best preserved on the right side of UMNH VP 16800, which possesses seven marginal undulations in addition to a seventh locus on the caudal midline. A caudomedian epiparietal is otherwise present only in the centrosaurine *Avaceratops* [21] and the chasmosaurines *Arrhinoceratops brachyops, Torosaurus latus, Torosaurus utahensis* and *Triceratops*.

The parietal transverse bar and lateral rami of *Nasutoceratops* are relatively thin (4–19 mm), even along the outer margins. Surface bone texture on this element appears to exhibit a mosaic of striated and mottled types, associated with subadult and adult status, respectively [22]. Although it is conceivable that the relatively unornamented frill of *Nasutoceratops* reflects an ontogenetic stage rather than the mature condition, other aspects of the specimen (e.g. fused vertebral centra and neural arches, epiparietals fused to marginal undulations) are indicative of adult status. Moreover, given that the epiparietals show no indication even of incipient hypertrophy, the frill morphology is postulated here to approximate the mature condition. Finally, the size of both UMNH VP 16800 and UMNH VP 19466 are consistent with the absolutely largest *Centrosaurus* skulls and the presence of adult bone texture [22,23] on the frill are consistent with an interpretation of adult status.

## 4. Discussion

Until recently, the lack of centrosaurine remains discovered in the American southwest prompted some investigators to postulate their existence only in the northern region of the palaeolandmass Laramidia [24]. *Diabloceratops*, from the early Campanian Wahweap Formation of Grand Staircase-Escalante National Monument, was the first named southern Laramidian exception [3]. *Nasutoceratops*, from the overlying Kaiparowits Formation, represents the second example, as well as the first southern centrosaurine from the late Campanian.

**Figure 2.**
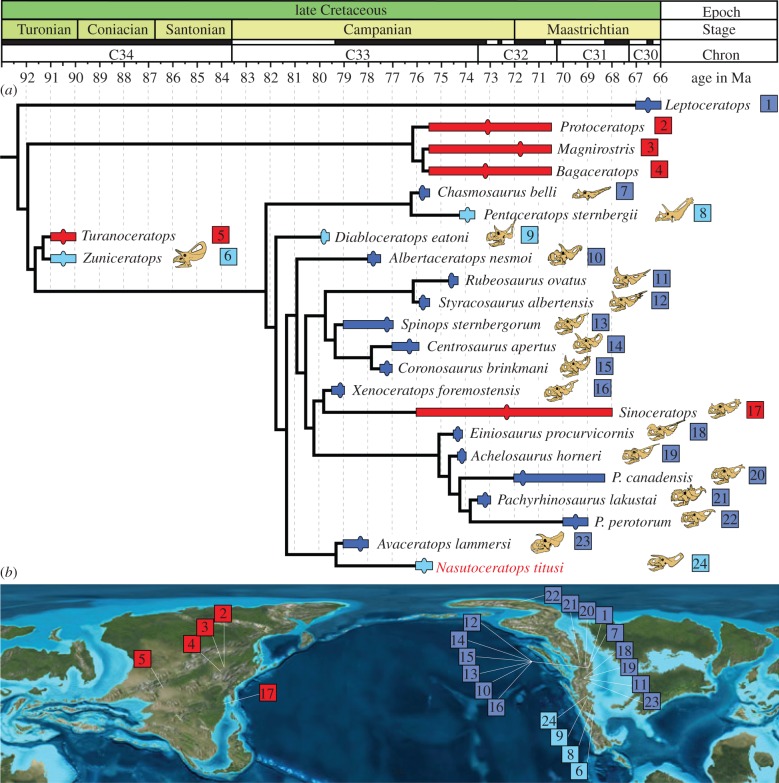
Time-calibrated phylogenetic relationships of *Nasutoceratops titusi*, n. gen et. sp. within Ceratopsidae (*a*). Single most parsimonious tree (tree length = 140, CI = 0.721, RI = 0.852) of an analysis of 97 characters. Species durations (bars) based on maximum and minimum stratigraphic occurrences correlated where possible to radiometric dates. Ovals in the ranges are either centroids or are the most likely age of taxa based on stratigraphic placement. Taxa listed in red represent Asian occurrences, those in dark blue represent northern Laramidia and those in light blue represent southern Laramidia. Stratigraphic data recorrelated and recalibrated from Sampson & Loewen [26] and Roberts *et al*. [17]. A further explanation for stratigraphic placement is presented in the electronic supplementary material. Occurrences of taxa presented in phylogeny placed on a Late Cretaceous palaeogeographic map (*b*) modified after Blakey [27].

In order to assess the phylogenetic position of *Nasutoceratops*, we added the scorings for this taxon into the matrix used for two recently published centrosaurines [20,25]; see the electronic supplementary material). *Nasutoceratops* can be confidently placed within Centrosaurinae on the basis of a suite of synapomorphies, including: premaxilla with pronounced ventral angle; subcircular narial region; presence of narial spine composed of nasal and premaxilla and rostrocaudally abbreviated squamosal with stepped caudomedian margin. However, despite its late Campanian age, approximately coeval with the highly derived northern taxa *Styracosaurus* and *Centrosaurus*, *Nasutoceratops* retains several primitive features (e.g. ventrally displaced maxillary toothrow, elongate supraorbital horncores, pronounced epijugals) otherwise absent in all other late Campanian centrosaurines. Phylogenetic analysis (figure 2; see also the electronic supplementary material for character definitions, matrix and expanded summary of results) places *Nasutoceratops* as the sister taxon of *Avaceratops* from the Judith River Formation of Montana [21,28]. Together, *Nasutoceratops* and *Avaceratops* form a previously unrecognized clade that branched off near the base of Centrosaurinae. Both possess secondarily simplified frills lacking prominent ornamentation of any epiparietal loci. Both lack a median embayment of the caudal midline of the parietal and instead possess a median undulation. Our understanding of centrosaurine evolution has increased dramatically in recent years, with 12 of the 17 currently known taxa described in the past decade alone. The emerging picture indicates that centrosaurines (and ceratopsids) originated on Laramidia 90–80 Myr ago. Early forms such as *Diabloceratops* possessed diminutive nasal horncores, relatively elongate supraorbital horncores and frills adorned with a single pair of elaborate marginal ornamentations. The present study reveals a basal split that resulted in two clades, both of which persisted into the late Campanian.

One of these clades, currently known only from northern Laramidia, evolved more elaborate frills with multiple hypertrophied epiparietals per side. Whereas middle Campanian representatives of this clade, such as *Diabloceratops* and *Albertaceratops*, retained diminutive nasal horncores and elongate supraorbital horncores, beginning approximately 77.5 Ma descendant taxa possessed elongate nasal horncores, abbreviated supraorbital horncores, and typically more elaborate frills. *Spinops sternbergii* is the oldest known member of the replacement assemblage, with subsequent taxa including *Coronosaurus*, *Centrosaurus*, *Styracosaurus* and *Rubeosaurus*. The most derived members of this clade are characterized by the possession of relatively low, thickened bosses in place of the skull roof horncores, as well as similarly ornate frills (i.e. *Achelousaurus* and *Pachyrhinosaurus*).

**Figure 3.**
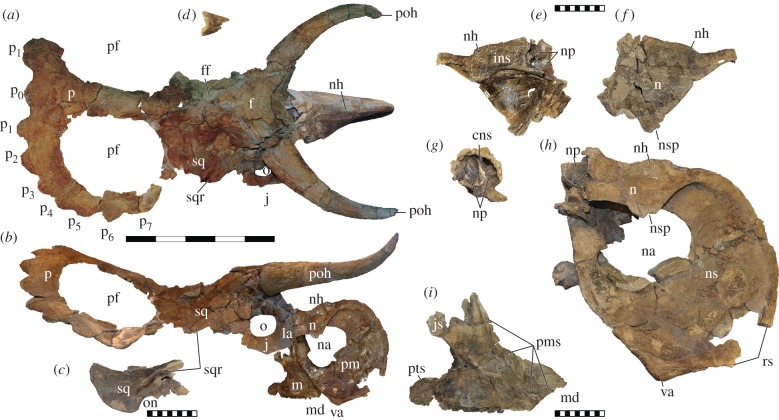
*Nasutoceratops titusi*, n. gen et. sp., holotype skull (UMNH VP 16800) in dorsal (*a*) and lateral (*b*) views. Scale bar represents 50 cm and the naris and maxilla are photoreversed in (*b*). Referred squamosal (UMNH VP 19469) in lateral view (*c*). Note lateral squamosal ridge in (*b*) and (*c*). Epijugal of the holotype (UMNH VP 16800) in anterior view (*d*). Nasal of referred skull (UMNH VP 19466) in medial (*e*) and lateral (*f*) view. Nasal (*g,h*), premaxilla (*h*), and maxilla (*i*) of the holotype in caudal (*g*) and lateral (*i*) views (photoreversed). Scale bars for (*c*–*i*) represent 10 cm. Abbreviations (autapomorphies noted with an asterisk (*)): cns, caudal nasal septum*; f, frontal; ff, frontoparietal fontanelle; ins, internarial suture; j, jugal; js, jugal suture; la, lacrimal; m, maxilla; md, maxillary diastema; n, nasal; na, naris; nh, nasal horncore; np, nasal pneumatopore*; ns, narial septum; nsp, narial spine; o, orbit; on, otic notch; p, parietal; p_0_–p_7_, epiparietals; pf, parietal fenestra; epiparietals; pm, premaxilla; pms, premaxillary suture*; poh, postorbital horncore*; pts, pterygoid-epipterygoid suture; rs, rostral suture; sq, squamosal; sqr, lateral squamosal ridge; va, ventral angle.

The second centrosaurine clade, identified here for the first time, to our knowledge, appears to have followed a distinctly different evolutionary trajectory, retaining relatively short nasal horncores and elongate supraorbital horncores, but simplifying the frill. This pattern of evolutionary modification parallels that within chasmosaurines, which also appear to have de-emphasized frill ornamentation in favour of enlarged supraorbital horncores. The early Campanian *Avaceratops* is the earliest known member of this second centrosaurine clade. *Nasutoceratops*, from the late Campanian of Utah, post-dating *Avaceratops* by approximately 2 Ma, is the latest-occurring form (figure 3). Together, these taxa demonstrate that this clade occurred in northern and southern Laramidia.

Several hypotheses have been put forth regarding the function of ceratopsid horn and frill structures, but the consensus view of the past several decades is that they functioned in intraspecific signalling, with horns also used in combat with conspecifics [29,30]. Recent debate has focused on two signalling alternatives, species recognition and mate competition, driven by natural and sexual selection, respectively [31–33]. Whatever the signalling function, evolutionary change within the two centrosaurine clades noted earlier was concentrated in different regions of the skull roof. Whereas the *Avaceratops*–*Nasutoceratops* clade secondarily reduced frill ornamentations and elaborated the supraorbital horns, the *Spinops*–*Pachyrhinosaurus* clade secondarily reduced supraorbital horns while hypertrophying both the nasal horn and frill ornamentation, effectively distributing bony apomorphic signalling structures across the entire skull roof.

*Nasutoceratops* provides additional support for the dinosaur provincialism hypothesis—the idea that distinct, coeval, latitudinally arrayed communities of theropods and ornithischians existed on Laramidia for more than 1 Myr of late Campanian time [11,12,24,34]. Although the northern taxon *Avaceratops* is the sister of *Nasutoceratops*, the former occurs in sediments that precede the latter by several million years. By the time of *Nasutoceratops*, northern centrosaurines all belonged to a distinct clade with more elaborate frill ornamentations. Thus, in addition to representing a previously unknown taxon not found in northern Laramidia, the discovery of *Nasutoceratops* suggests that a distinct, persistent, previously unrecognized clade of centrosaurines inhabited southern Laramidia during this interval.

Considered in unison, four lines of evidences suggest that *Nasutoceratops* represents the first example of a previously unknown Campanian radiation of southern Laramidian centrosaurines bearing elongate supraorbital horns and simple frills. First, robust evidence now exists of distinct, highly diverse northern and southern vertebrate communities on Laramidia [11,12,34]. Second, among dinosaur clades, ceratopsids in particular underwent rapid evolutionary turnover during the Campanian [20,26], becoming the most diverse clade of Laramidian dinosaurs. Third, none of the 14 centrosaurine taxa known from northern Laramidia have been found in the south. Fourth, evidence is now accumulating that centrosaurines underwent substantial diversification in southern Laramidia early in the Campanian [35], and we know of no *a priori* reason to expect a substantially different pattern during the late Campanian, particularly given the many taxa unearthed in Alberta and Montana for this interval. In short, *Nasutoceratops* adds a critical element to a rapidly emerging evolutionary picture, offering the first glimpse into centrosaurine diversity on southern Laramidia during the Late Campanian.

## Data accessibility

The original character-taxon matrix and tree file are freely available on Morphobank as project 964 and matrix 2151 and TreeBASE as project 14321. See the electronic supplementary material for a complete description of the characters and specimens studied.
